# In the Interest of Race Integrity

**Published:** 1911-02

**Authors:** 


IN THE INTEREST OF RACE INTEGRITY.—The editor of this JOURNAL has prepared a bill to sterilize certain male lunatics when furloughed or discharged, and criminals in the penitentiary who have been twice or oftened convicted of felony,—*and to do more than vasectomise the rapist-by-force.* It was introduced into the House by Dr. Daniel Parker, of Calvert, February 1st, inst., and sent to the Committee on Public Health—all doctors but one, to-wit: Parker, Hubbard, Gross (Chairman), White, Smith and Goodner. It is framed after the Connecticut law. See Dr. Bogart's paper in this issue.

---

FIZZ.—A stuttering man named Sisson, arraigned before the magistrate, was asked his name. He replied, "Sis-sis-sis-*sizzen*" The judge, turning to the policeman, said: "What's this man charged with?" "Soda water, your honor," said the cop.

